# Cognitive biases in pediatric cardiac care

**DOI:** 10.3389/fcvm.2024.1423680

**Published:** 2024-07-04

**Authors:** Paul Padovani, Arnaud Roy, Amanda Guerra, Olivier Cadeau, Mohamed Ly, Corina M. Vasile, Robert H. Pass, Alban-Elouen Baruteau

**Affiliations:** ^1^ CHU Nantes, Department of Pediatric Cardiology and Pediatric Cardiac Surgery, FHU PRECICARE, Nantes Université, Nantes, France; ^2^ CHU Nantes, INSERM, CIC FEA 1413, Nantes Université, Nantes, France; ^3^ LPPL, SFR Confluences, Nantes Université, Université d’Angers, Angers, France; ^4^ CHU Nantes, Centre Référent des Troubles d’Apprentissage, Nantes Université, Nantes, France; ^5^ Pediatrics Department at Filantropia Municipal Hospital of Craiova, Craiova, Romania; ^6^ Department of Pediatric Cardiology, Mount Sinai Kravis Children’s Hospital, New York, NY, United States; ^7^ CHU Nantes, CNRS, INSERM, l’institut du Thorax, Nantes Université, Nantes, France; ^8^ INRAE, UMR 1280, PhAN, Nantes Université, Nantes, France

**Keywords:** congenital heart disease, cognitive biases, pediatric cardiology, diagnostic errors, medical decision-making, human factors

## Abstract

Medical practitioners are entrusted with the pivotal task of making optimal decisions in healthcare delivery. Despite rigorous training, our confidence in reasoning can fail when faced with pressures, uncertainties, urgencies, difficulties, and occasional errors. Day-to-day decisions rely on swift, intuitive cognitive processes known as heuristic or type 1 decision-making, which, while efficient in most scenarios, harbor inherent vulnerabilities leading to systematic errors. Cognitive biases receive limited explicit discussion during our training as junior doctors in the domain of paediatric cardiology. As pediatric cardiologists, we frequently confront emergencies necessitating rapid decision-making, while contending with the pressures of stress, fatigue, an earnest interest in “doing the right thing” and the impact of parental involvement. This article aims to describe cognitive biases in pediatric cardiology, highlighting their influence on therapeutic interventions for congenital heart disease. Whether future pediatric cardiologists or experienced professionals, understanding and actively combating cognitive biases are essential components of our ongoing medical education. Furthermore, it is our responsibility to thoroughly examine our own practices in our unwavering commitment to providing high-quality care.

## 1 Introduction

Type 1 processing, also known as intuitive decision-making or heuristic reasoning, refers to a rapid and automatic thinking process. It is characterized by the utilization of mental heuristics, or simple rules, to swiftly reach conclusions and make decisions without requiring in-depth analysis of available information (1). In addition to Type 1 processing, there exists Type 2 processing, characterized by slower, more deliberate analysis of information and consideration of multiple factors (2). Despite the essential nature of Type 1 processing (3), it can lead to systematic thinking errors, called cognitive biases. A cognitive bias is a systematic deviation from rationality or objectivity in judgment or decision-making, often stemming from mental shortcuts, perceptual distortions, or subjective influences. Importantly, these biases do not correlate with intelligence or cognitive ability (4, 5). They arise from a variety of sources including acquired or inherent predispositions, societal and cultural influences, deficits in statistical understanding and mathematical reasoning and, in particular, and environmental stimuli that demand our attention (6).

Cognitive biases can impact various facets of our existence, but their significance becomes pronounced when these errors manifest within the context of medical practice. Prevalence of diagnostic error has been estimated to be as high as 10%–15% in daily clinical practice (7). Cognitive factors are the main contributor to diagnostic errors, which are associated with a proportionately higher morbidity than is the case with other types of medical error (8, 9). Contrary to physician interviews that often identify system-related factors (organizational flaws, inadequate policies, staffing or equipment) as the main contributors to diagnostic errors, cognitive factors are more likely the primary driver of such errors (10).

Among medical specialties, paediatrics stands out as one where decisions are emotionally demanding, given the significant weight they carry for both parents and children (11, 12). Given the high stakes involved in pediatric care, understanding, and mitigating cognitive biases is paramount.

This work aims to examine the medical reasoning and practice by highlighting several cognitive biases specifically within the field of pediatric cardiology and congenital heart disease (CHD) care.

## 2 Subsections relevant for the subject

Cognitive biases are prevalent in pediatric cardiology practice and can significantly impact diagnostic decision-making, multidisciplinary collaboration, and technical procedures.

### 2.1 Diagnostic decision-making

*This section delves into cognitive biases that influence diagnostic decision-making such as the availability bias, the anchoring bias, the attrition bias, the confirmation bias, overconfidence, and search satisfying.*

Cognitive biases have been identified in all steps of decision making (13–15), including information gathering, association triggering, context formulation, processing and verification (16). The diagnostic enterprise, construed as the distinctive characterisation of a specific disease or condition, hinges on factors including etiopathogenesis, parental interrogation, children signs, symptoms, physical examination results, diagnostic tests, and health history. Clinical expertise accrues through domain-based practice, augmented experience, enhanced knowledge and skills, and the development of domain-specific intuitive capacities. In addition to the advanced cognitive functions such as problem-solving, judgment, and decision-making mentioned earlier, formulating a diagnosis also relies on the utilization of social and emotional resources. Contemporary understanding of cognition underscores the dynamic interaction between cognitive processes and socioemotional factors in decision-making contexts. This includes the impact of emotional states, interpersonal dynamics, and cultural influences on cognitive functioning (17, 18). Expert clinicians find it easier to effectively focus attention on and evaluate details of the infant's clinical problem. This enables them to intuitively and deliberately generate several relevant differential diagnoses and potential strategies to address the identified clinical situation.

**Figure 1:**
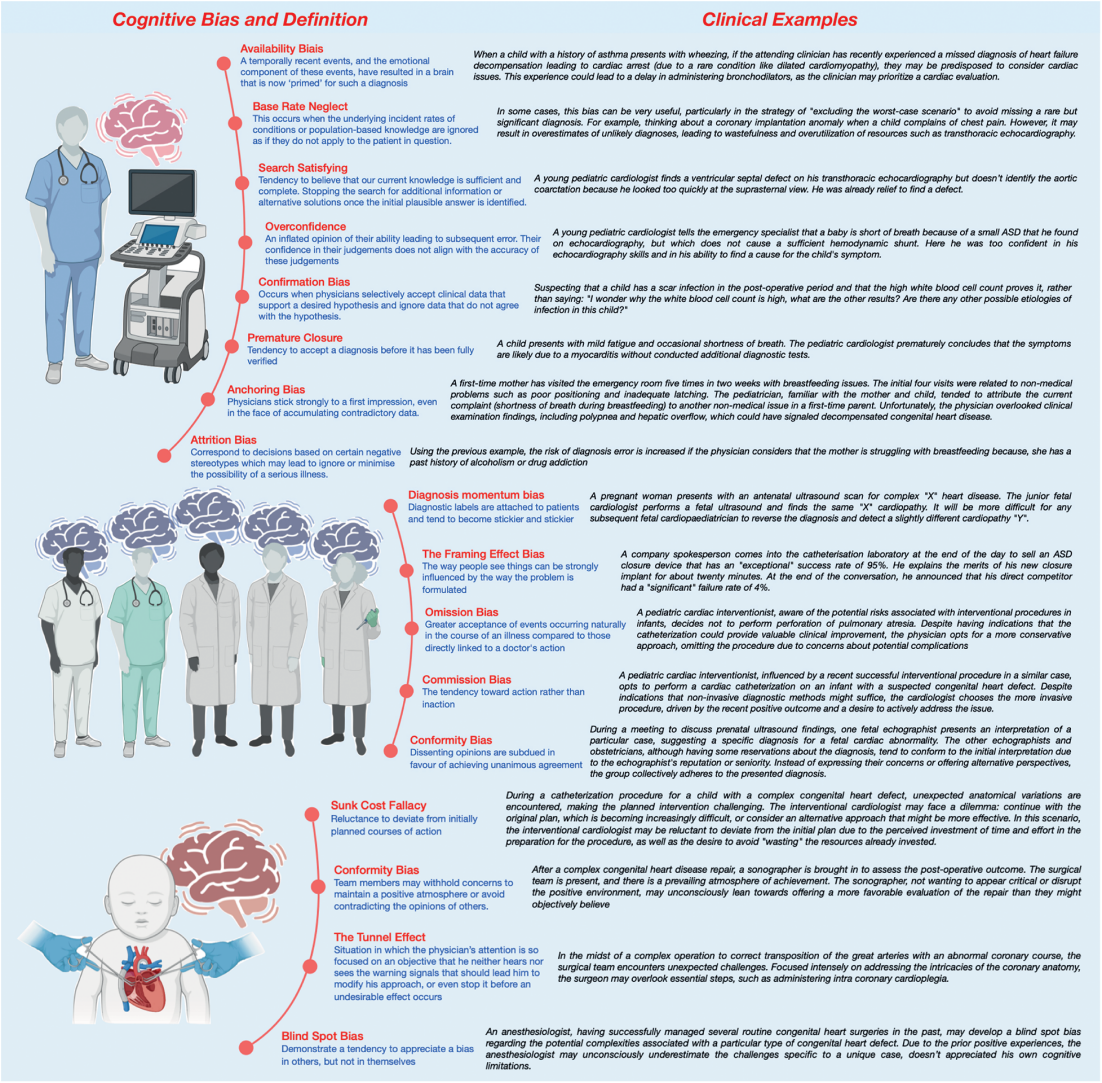
Clinical examples of cognitive biases during pediatric cardiac care.

Nonetheless, experience can be a source of bias, such as ***availability bias*** (19–21) or ***base rate neglect*** (22) (see Figure 1). Physicians often tend to perceive things as more likely if they readily come to mind: “common things are common (availability)”. They may form diagnostic hypotheses based on recent exposure to another patient with the same illness, particularly if the case was particularly notable or emotionally charged. Conversely, prolonged absence in encountering a disease tends to decrease the likelihood of considering it during diagnosis: “out of sight out of mind (non-availability)”. The ***bias of restricting representativeness*** is ingrained in the traditions of medical education (23). Our diagnostic approach often leads us to search for prototypical presentations of a disease: adhering to the adage “When you hear hoofbeats, think of horses rather than zebras”. These entrenched dogmas and mindsets tend to exclude rare diseases from consideration in the diagnostic process. This can be particularly detrimental in the field of CHD, which may present infrequently in emergency room consultations despite being relatively common, affecting 8 out of 1,000 births (24). ***Base rate neglect*** on the other hand may result in overestimates of unlikely diagnoses. This bias occurs when individuals fail to consider statistical or base rates in decision-making, instead prioritizing specific information or individual cases.

Another potential bias, notably observed in emergency departments (ED), is ***anchoring bias*** whereby physicians may strongly adhere to their initial impressions, even when confronted with a substantial amount of conflicting data, particularly if they harbor certain preconceptions (23, 25). Anchoring bias is linked to ***attrition bias*** which is attempts to discover reason for observations (26). Stereotyping and gender bias are both good examples of attrition bias. In the ED, there is often significant time pressure to make swift decisions. This urgency is particularly concerning in the context of undiagnosed CHD, where achieving accurate diagnoses may necessitate thorough and careful consideration in addition to multiple examinations such as electrocardiography, chest radiographs, echocardiography, and blood tests.

Compared with an expert, novice specialists will likely make more diagnostic errors (18). Formulating a specific diagnosis largely relies on pattern recognition, the clinician's familiarity with similar clinical scenarios, and the ease with which relevant past cases come to mind. Confirmation Bias, Overconfidence and Search Satisfying are common bias for novice specialists (27). ***Confirmation bias***, the selective acceptance of clinical data supporting a desired hypothesis while disregarding contradictory data, significantly heightens the risk of diagnostic errors (28). ***Overconfidence***, also known as the Dunning-Kruger effect, describes the tendency for individuals lacking expertise in a given field to overestimate their proficiency, leading them to believe they possess more knowledge than they actually do (29, 30). This can lead to noncompliance with evidence-based guidelines. Additionally, ***search satisfying*** occurs when clinicians cease seeking additional information once they find a solution that appears satisfactory, even if it may not be the most appropriate or accurate (14). Unlike confirmation bias, where clinicians tend to seek evidence to confirm their initial hypotheses while ignoring contradictory evidence, search satisfying focuses more on the satisfaction of finding a plausible solution, even if it is not validated by additional evidence or thorough consideration. Paradoxically, qualified individuals tend to underestimate their own abilities. In other words and restated, inexperience may prevent people from recognising their own limitations and those who are more highly qualified and experienced may paradoxically underestimate the extent of their knowledge.

Another well-known bias is ***premature closure*** which is a form of bias in which we tend to be satisfied with a plausible hypothesis derived from our own experience and dismiss the possibility of uncertainty (31). Moreover, when contemplating the dynamics of the doctor-parent relationship, particularly within the nuanced context of paediatrics, a salient cognitive bias that warrants consideration is the ***outcome bias***. This cognitive inclination manifests as a predisposition towards diagnostic decisions that are anticipated to yield positive outcomes, consciously avoiding choices associated with unfavourable consequences (32). Such a cognitive predisposition serves as a protective mechanism, strategically sidestepping the emotional distress that may be entwined with less favourable clinical outcomes. This can lead to minimizing serious diagnoses or hoping for a favourable outcome and may manifest, in pediatric cardiovascular care as the avoidance of surgery or a catheterization procedure, as two examples. Also, ***countertransference*** (positive or negative) can lead to an under-assessment of the severity of the child or adolescent's condition, to avoid breaking bad news (33, 34). In an ideal scenario, every clinical decision would be made impartially and consistently across different patients. However, this isn't always the case. Our interactions with patients and families can evoke both favorable and unfavorable sentiments, which might influence the quality of our decisions. Within the context of the ED, instances may arise where a physician experiences positive countertransference towards a patient, potentially exerting an influence on clinical decision-making processes. This phenomenon, driven by outcome bias, could lead to underinvestigation, as decisions favoring positive outcomes may take precedence over those indicating negative outcomes. Consequently, this bias may result in the omission of diagnostic tests critical for identifying unfavorable prognostic implications for the patient.

Added to and closely linked to cognitive biases is the notion of noise which reflects errors in judgement and measurement. Some situations are more prone to biased-reasoning due to this form of error (17). The profound influence of background noise on decision-making, occasionally impeding the activation of System 2 cognition—the reflective and analytical mode of thought—is highlighted by factors including workload overload, concurrent multitasking, task interruptions, fatigue, time pressure, the broader work environment, dysfunctional team dynamics, hyperconnectivity, and various forms of distraction. These distractions may arise from external sources or be influenced by temporal factors such as the day of the week, holidays, or the time of day or night. A constant self-inquiry that warrants consideration is whether the current situation is conducive to the prevalence of biases.

### 2.2 Multidisciplinary collaboration

*Exploring cognitive biases during multidisciplinary decision-making highlights challenges and opportunities for enhancing teamwork. These biases include memory shifting/reconstruction bias, diagnosis momentum bias, framing effect bias, order effect bias, bias of omission, commission bias, conformity bias, authority gradient effect, and hindsight bias*

Multidisciplinary consultations and collaborative thinking represent effective strategies for mitigating decisions influenced by rapid reasoning with cognitive biases (35–38) (see Figure 1). However, certain inter-human factors may still be biased and affect such decision-making. ***Memory shifting***, also called ***reconstruction bias***, involves the inaccurate recall of information due to variations in coding of meaning and textual information, resulting in the “filling in” of details, sometimes with incorrect information, during memory recall. ***Diagnosis momentum*** bias is another concern, whereby diagnostic labels attached to patients tend to become increasingly ingrained over time, leading to the exclusion of alternative possibilities. This bias is closely related to the ***framing effect bias***, which demonstrates how the formulation of a problem can strongly influence perception (39). Additionally, ***the order effect bias*** must be considered when presenting cases, as individuals tend to remember the beginning (primacy effect) or the end (recency effect) of a story being told, highlighting the importance of how cases are presented in such conferences (13, 17). This holds particularly true when presenting a case of a child who has undergone multiple surgeries for a complex cardiac disease. The order in which the varied problems and interventions have been presented can have a meaningful and unintentional impact on how the case is discussed and considered.

In medical decision-making, there is a tendency toward inaction, prioritizing the principle of non-maleficence, which leads to the ***omission bias*** (32, 36, 40). This bias, influenced by the perceived safety of inaction, can have severe consequences despite maintaining the status quo. Conversely, ***commission bias***, stemming from the obligation toward beneficence, involves a propensity toward action. This bias is more prevalent in overconfident physicians. Discussing these two biases in the context of pediatrics is particularly important, given the unique ethical considerations and potential consequences of either form of bias on the care of pediatric patients.

Moreover, within the intricate dynamics of medical staff discussions, the susceptibility to ***groupthink or false consensus effect*** poses a significant challenge (36). The desire for consensus may lead to a ***conformity bias,*** wherein dissenting opinions are subdued in favour of achieving unanimous agreement. This can be particularly pronounced in hierarchical medical teams, where junior members may hesitate to challenge prevailing views. Specifically, the phenomenon referred to as the ***authority gradient effect*** comes into play in such situations. The authority gradient effect describes the reluctance of junior members to challenge the opinions of senior members within hierarchical structures. Furthermore, the phenomenon of ***hindsight bias*** whereby there may be a tendency to perceive something as having been more predictable (e.g., “I knew it all along”) than it truly is while making decisions (41). This retrospective distortion may impact how post-case analyses unfold and potentially impede the recognition of avoidable errors. These behaviours may be conscious and induced by social norms, but often, they are unconscious and characteristic of cognitive biases.

### 2.3 Technical procedure

*Exploring cognitive biases during technical procedures is essential for identifying potential errors and implementing risk-mitigation strategies. Anesthesiologists, surgeons, and cardiac interventionists, encounter various cognitive biases during medical procedures such as anchoring bias, sunk cost fallacy, social desirability bias and the tunnel effect.*

Anesthesiologists confront various cognitive biases that can significantly impact decision-making (6, 39). ***Anchoring bias—***the tendency to fixate on specific features -, for instance, becomes pronounced when faced with unexpected challenges during surgery, including hemodynamic variation. For example, anchoring bias becomes prominent in such situations. The initial medication dosage administered acts as a cognitive anchor, disproportionately influencing subsequent decisions. This predisposition may lead to either inadequate or excessive adjustments.

Confronted with unexpected challenges in the operating room or the catheterisation laboratory, physicians (cardiac interventionists, surgeons, or anaesthesiologists) may grapple with the ***sunk cost fallacy***, fostering a reluctance to deviate from initially planned courses of action (42). This psychological bias arises from a perceived investment of time and effort, impeding objective reassessment. Additionally, the dynamics of communication within the anesthesia/nurse/surgery team may exhibit a ***conformity bias***, wherein team members may withhold concerns to maintain a positive atmosphere or avoid contradicting the opinions of others. Of course, physicians during technical care are also vulnerable to many of the previously described biases such as confirmation bias or overconfidence bias amongst others.

Surgeons and cardiac interventionists are highly sensitive to the ***tunnel effect*** (42, 43). This refers to a situation in which the physician's attention is so focused on an objective (expected outcome, management of a complication) that he neither hears nor sees the warning signals that should lead him to modify his approach, or even stop it before an undesirable effect occurs. In addition, proceduralists must deal with a variable range of emotions that may influence their decision-making during procedures. For example, anger can influence decisions made by oneself or the team, regret describes the tendency to let regret about past decisions influence future decisions and anticipatory regret is the desire to avoid regret about future consequences or outcomes of decision choices. All of these may negatively impact the proceduralist.

Finally, and somewhat paradoxically, the well-known ***blind spot bias***, whereby individuals tend to recognize biases in others but not in themselves, is observed (15, 17, 36). A blind spot bias may occur when a physician, while evaluating a child with cardiac symptoms, readily identifies and corrects reasoning errors in their colleagues but fails to question their own diagnostic decisions or treatments, even if they are affected by their own similar cognitive biases.

## 3 Discussion

Identifying physicians’ cognitive biases at an early stage is essential for optimising medical decisions, preventing errors and creating realistic expectations for patients, ultimately reducing the rising costs of healthcare (44). Most cognitive biases probably arise from overuse of System 1 or when System 1 dominates over System 2. Techniques that enhance System 2 could counteract these biases, thereby improving diagnostic accuracy and decreasing management errors (45).

Overconfidence, anchoring bias, and availability bias were prevalent, impacting diagnostic accuracy in 36.5%–77% of case scenarios (46). For example, Mamede et al. found that availability bias increased with years of training, and reflective reasoning improved diagnostic accuracy among internal medicine residents (21). Additionally, biases such as information bias, representativeness bias, and premature closure were associated with diagnostic errors in over half of the evaluated scenarios. These findings highlight the widespread presence of cognitive biases across various medical tasks, from diagnosis to treatment and management (46).

Cognitive biases not only affect diagnostic accuracy but also influence therapeutic and management decisions. For instance, Yee et al. found that better-coping strategies and higher tolerance to ambiguity among obstetricians were associated with lower rates of instrumental vaginal deliveries and fewer management errors (47). They also indicated that higher tolerance to ambiguity among physicians was associated with increased medical complications, such as postpartum hemorrhage. These studies demonstrate how cognitive biases can lead to both over-treatment and under-treatment, illustrating the necessity for balanced decision-making frameworks. This also underscores the potential severity of cognitive biases, emphasizing the need for further research to establish clear links between these biases and patient outcomes (48).

Addressing cognitive biases requires a multi-faceted approach. Increasing awareness among physicians and medical students is crucial, as is incorporating training on cognitive biases into medical education programs. Effective strategies include reflective reasoning, the use of cognitive checklists, and heuristic approaches to simplify decision-making processes. Collaborative efforts from academic institutions, healthcare organizations, and policymakers are needed to implement these strategies and improve healthcare delivery (49–51).

## 4 Conclusion

A significant portion of today's medical decision-making research evolved from studies conducted in the field of cognitive psychology in the late 20th century (52). Regrettably, despite this wealth of accumulated knowledge, medical practitioners still struggle to fully comprehend and integrate these findings into their practice. Grounded in real-world examples and informed by personal experiences, this perspective endeavors to elucidate the intricate relationship between cognitive biases and the landscape of pediatric cardiology. However, this work represents only an initial step toward grasping the complexities of human decision-making dynamics. There is a notable lack of comprehensive research in this arena in pediatrics, prompting critical inquiry into the interaction between cognitive biases and the powerful stresses and emotions experienced in pediatric care. To address these gaps, further research is imperative, along with the development of dedicated protocols and frameworks to help mitigate the impact of these biases on decision-making. Incorporating training or simulations in human factors, including the study of cognitive biases, into the curriculum of young pediatricians across all specialties should be considered as an essential component for enhancing clinical practice and patient care.
